# Does a temporizing measure of cerebrospinal fluid drainage as the initial procedure alter the surgical outcome in premature infants with post-hemorrhagic hydrocephalus?

**DOI:** 10.1186/2045-8118-12-S1-P8

**Published:** 2015-09-18

**Authors:** Eisha Anne Christian, Edward Melamed, Edwin Peck, Mark D Krieger, J Gordon McComb

**Affiliations:** 1 University of Southern California, Los Angeles, CA, USA; 2 Children's Hospital Los Angeles, CA, USA

## Objective

It has been speculated whether the insertion of a temporary device to control hydrocephalus secondary to intraventricular hemorrhage (IVH) in the preterm neonate with removal of the debris caused by such a hemorrhage, can reduce subsequent complications following insertion of a permanent cerebrospinal fluid (CSF) diverting shunt. This retrospective review is directed at examining this speculation.

## Methods

A retrospective review of the medical records of all premature infants surgically treated for post-hemorrhagic hydrocephalus (PHH) between 1997 and 2012 at our institution was undertaken.

## Results

Over 14 years, 91 preterm infants with PHH were identified. The initial procedure for 50 neonates was the insertion of a ventricular reservoir (VR) that was serially tapped for varying time periods. For the remaining 41 premature infants, a ventriculoperitoneal/atrial shunt (VS) was the first procedure. Patients with a VR as their initial procedure underwent CSF diversion significantly earlier in life than those who had VS as the initial procedure (29 vs. 56 days, p < 0.01). Of the infants with a VR as their initial procedure, 5/50 (10%) did not undergo a subsequent VS. The number of shunt revisions and the rates of loculated hydrocephalus and shunt infection did not statistically differ between the two groups.

## Conclusion

Patients with initial VR insertion received a CSF diversion procedure at a significantly younger age than those who received a permanent shunt as their initial procedure. Otherwise, the outcomes with regards to shunt revisions, loculated hydrocephalus, and shunt infection were not different for the two groups.
